# The Radiobiological Effect of Flattening Filter Free (FFF) Beam Delivery Parameters on A549 and MCF-7 Cell Survival: An In Vitro Study

**DOI:** 10.3390/life16060903

**Published:** 2026-05-27

**Authors:** Cemile Ceylan, Kamil Can Kılıç, Ahmet Öztürk, Canan Öztürk, Yusufhan Yazır, Maksut Görkem Aksu, Özcan Gündoğdu

**Affiliations:** 1 Department of Radiation Oncology, Medicalpark Istanbul Oncology Hospital, 34180 Istanbul, Türkiye; 2 Department of Medical Physics, Yeditepe University, 34755 Istanbul, Türkiye; 3 Department of Stem Cell, Institute of Health Sciences, Kocaeli University, 41380 Kocaeli, Türkiye; k.kilic03@gmail.com (K.C.K.); ahmet.ztrk@icloud.com (A.Ö.); canantugozturk@gmail.com (C.Ö.); yusufhanyazir@yahoo.com (Y.Y.); 4 Center for Stem Cell and Gene Therapies Research and Practice, Kocaeli University, 41380 Kocaeli, Türkiye; 5 Department of Histology and Embryology, Faculty of Medicine, Kocaeli University, 41380 Kocaeli, Türkiye; 6 Department of Radiation Oncology, Faculty of Medicine, Kocaeli University, 41380 Kocaeli, Türkiye; aksugorkem@kocaeli.edu.tr; 7 Department of Biomedical Engineering, Faculty of Technology, Kocaeli University, 41380 Kocaeli, Türkiye; o.gundogdu@kocaeli.edu.tr

**Keywords:** A549, MCF-7, clonogenic assay, flattening filter-free, radiosensitivity, dose rate, dose per pulse

## Abstract

Background: Recent improvements, such as flattening filter-free (FFF) beams, enable the delivery of treatments at higher dose rates and in shorter times. However, the biological influence still remains uncertain. Here, we examined how dose rate and dose per pulse influence responses to FFF beams compared with flattened beams in cancer cells with different radiosensitivities. Methods: A549 (lung, high α/β) and MCF-7 (breast, low α/β) cells were irradiated using VMAT (6 MV FF, 6 and 10 MV FFF; 600–2400 MU/min; 5–20 Gy). Cell viability was assessed by the WST-1 assay, and reproductive survival by clonogenic assay. Results: The responses differed by cell type and dose, with A549 exhibiting restricted sensitivity, demonstrating more pronounced effects at 10 Gy, while MCF-7 displayed greater responsiveness at 5–10 Gy under elevated dose rates. At 20 Gy, differences were reduced. Metabolic readouts did not consistently match clonogenic survival. Conclusions**:** Overall, FFF effects appear cell-specific, and the suggestion is that clinical translation should be guided by tumor-specific radiosensitivity rather than an assumption of universal benefit.

## 1. Introduction

The evolution of the linear accelerator (LINAC) technology has markedly enhanced the precision, efficiency, and overall effectiveness of radiation therapy (RT), particularly through the clinical implementation of flattening filter-free (FFF) photon beams. Traditional Linac systems employ a flattening filter to produce a uniform dose distribution across the treatment field; however, this component also limits the achievable dose rate and contributes to beam hardening and increased head scatter. The removal of the flattening filter in FFF configurations eliminates these limitations, resulting in significantly higher instantaneous dose rates and a more intense central beam profile. Consequently, FFF beams substantially reduce beam-on time while maintaining, and in some cases improving, dosimetric quality and therapeutic efficacy. This advancement has been particularly impactful in high-dose hypofractionated treatment protocols, such as stereotactic body radiation therapy (SBRT), where treatment regimens commonly involve single- or few-fraction deliveries with high doses per session ranging from 8 to 24 Gy [1]. For example, 6 MV FFF beams can deliver dose rates at the central axis of up to 1400 MU/min, which is more than twice the 600 MU/min of conventional flattened 6 MV beams. Similarly, the 10 MV FFF beams reached 2400 MU/min. The resulting reduction in beam-on time of up to 40% offers several clinical advantages. Shorter treatment sessions decrease patient discomfort, particularly in cases requiring long immobilization periods, and reduce the risk of patient movement during treatment, thereby improving targeting accuracy. In addition, increased treatment efficiency enhances patient throughput, allowing more patients to benefit from advanced RT techniques. These improvements underscore the growing role of FFF beam technology in modern radiation oncology and set the stage for further exploration of its clinical advantages and limitations.

Recent investigations have demonstrated that stereotactic radiosurgery techniques employing both flattening-filter radiotherapy (FF-RT) and flattening-filter-free radiotherapy (FFF-RT) can induce direct tumor cell apoptosis and vascular disruption owing to the typically high dose per fraction administered [2,3]. However, the clinical application and radiobiological efficacy of FFF beams remain contentious, as preclinical studies have shown considerable variability in outcomes. Our previous research on prostate cancer cell lines, along with studies by others, revealed divergent responses to FFF-RT [4]. Some investigations have reported comparable levels of cell survival between conventional dose-rate FF and high-dose-rate FFF exposure in both normal and cancerous cell populations [5,6,7,8,9]. While several studies have observed no significant difference in in vitro clonogenic survival following FFF or FF irradiation, emerging evidence suggests the potential advantages of high-dose-rate FFF beams in specific conditions. It has been demonstrated that glioblastoma cell lines exhibit a more pronounced decrease in clonogenic survival when exposed to FFF beams [10]. Similarly, scientists have reported enhanced antitumor effects under hypoxic conditions following the application of FFF beams [11]. Notably, both FF-RT and FFF-RT modalities induce apoptosis as the primary mechanism of tumor cell death; however, the capacity of FFF to deliver higher instantaneous dose rates has been associated with a substantial reduction in beam-on time, an operational benefit that may also influence biological responses [12].

Research has indicated that the survival dynamics of diverse cell groups, such as those present in most cancerous tumors, are dictated by specific linear-quadratic (LQ) survival models that differ according to the range of total radiation doses [13]. At lower doses (typically <3–5 Gy) and higher doses (>7–9 Gy), cell survival may follow distinct LQ-associated response patterns, whereas intermediate-dose regions can deviate from the classical linear-quadratic model. These variations may influence α/β ratios and biologically effective dose (BED) estimations, reflecting the complexity of tumor and normal tissue responses to ionizing radiation. In addition to total dose, radiation response is also influenced by fraction size, dose delivery conditions, and overall treatment time. The linear-quadratic (LQ) framework captures intrinsic radiosensitivity via two parameters: alpha (α; single-event lethality) and beta (β; interaction of sublethal lesions), and summarizes fraction-size sensitivity by the ratio α/β (the dose at which α and β contributions are equal). Systems with higher α/β are less sensitive to changes in fraction size (more “acute-like”), whereas lower α/β implies greater fraction-size sensitivity (more “late-like”) [14]. This complexity also extends to early and late radiation-induced toxicities, where the α/β ratios relevant to acute and chronic injuries are dose-dependent and may not be consistent across different dose regimens. Although the biological mechanisms underlying radiation-induced cell death are not fully understood, apoptosis and mitotic catastrophe are recognized as the two predominant mechanisms [15,16]. Malignant tissues have been assumed to possess high α/β ratios; however, recent radiobiological evidence has shown that several tumors, including breast and prostate cancers, may exhibit relatively low α/β ratios and greater sensitivity to hypofractionated irradiation [17,18]. The development of predictive assays capable of determining tumor-specific α/β ratios prior to treatment could enable more personalized radiotherapy protocols, optimizing dose fractionation to maximize therapeutic benefits while minimizing toxicity.

Recent studies have explored the radiobiological effects of high instantaneous dose rates delivered by FFF LINACs, particularly in clonogenic cell survival [19,20]. Although it has been reported that the instantaneous dose rate has no significant effect on survival outcomes [21], other studies have suggested that the radiobiological efficacy of FFF beams may be influenced by the dose per pulse (DPP), indicating that this parameter could be a critical determinant of the tumor cell response [10,22]. In FFF beams, the removal of the flattening filter leads to a marked increase in both the dose rate and DPP, which shortens the treatment times and alters the radiobiological profile of the beam. An elevated DPP means that more radiation is delivered in each pulse, potentially reducing the opportunity for sublethal damage repair between pulses and thereby enhancing tumor cell kill. Additionally, the higher overall dose rate in FFF beams may improve treatment precision by minimizing intrafraction motion. While these characteristics offer potential therapeutic advantages, the biological effects of increased DPP and dose rate are not yet fully understood and may vary depending on the tumor type, total dose, and fractionation scheme. Furthermore, scientists have emphasized that the overall beam-on time, rather than the average or instantaneous dose rate, is the key factor influencing tumor cell survival following external-beam radiotherapy [5]. Despite technological advances in radiotherapy, acquired tumor radioresistance remains a significant barrier to effective treatment. The underlying mechanisms of this resistance have yet to be fully elucidated, necessitating further investigation into the factors that modulate cellular radiosensitivity. In our study, we investigated the radiobiological impact of increasing the DPP under two distinct planning conditions. First, we increased the DPP while maintaining a constant average dose rate and achieved approximately the same monitor unit (MU) value, thereby primarily exploring the contribution of DPP within the applied delivery configuration. In a separate experimental setup, we further elevated the DPP by increasing the dose rate from 600 to 1400 MU/min for the same photon energy while keeping the total MU value constant. These approaches allowed us to explore delivery-configuration-associated radiobiological responses related to DPP and dose rate alterations under clinically relevant irradiation conditions. The α/β ratio and corresponding BED calculations are known to differ between low- and high-dose ranges in malignant tissues, which further complicates treatment optimization. Although dose-rate sparing-reduced biological response at lower dose rates-is a well-established concept [23], an “inverse dose-rate effect” has been described in tumor cells, where a lower dose results in decreased survival compared with higher rates [24]. Similar findings have been reported, indicating that increased dose rates do not uniformly enhance cell death and may, in some cases, attenuate therapeutic efficacy [25]. In the current study, we investigated the biological effects of varying dose rates, including 14, 6, and 24 Gy/min, using 6 MV FF, 6 MV FFF, and 10 MV FFF X-rays from a clinical LINAC, focusing on two cancer cell lines: A549 human lung carcinoma and MCF-7 human breast adenocarcinoma cells. Owing to their differential radiosensitivity, these cell lines were selected to explore the effects of irradiation with varying fractionation schemes; however, the use of FFF beams remains uncommon in routine clinical practice, warranting further investigation. To our knowledge, this was the first evaluation of the FFF beam dose-rate effects on the clonogenic survival of these specific cell models. Our findings contributed to a growing body of evidence highlighting the complexity of dose-rate responses in cancer radiobiology and may offer insights into the optimization of advanced radiotherapy modalities, including intensity-modulated radiation therapy (IMRT) and Volumetric Modulated Arc Therapy (VMAT). Although some earlier studies did not identify notable in vitro differences between FFF and FF beams, other studies have indicated that FFF beams may boost antitumor effects, especially in hypoxic and glioblastoma cell environments. By comparing clonogenic survival across different dose rate settings, we aimed to investigate whether the dose-rate sensitivity in FFF beam delivery was influenced by intrinsic cellular radiosensitivity.

## 2. Materials and Methods

### 2.1. Cell Culture

The experimental setup utilized two distinct human cancer cell lines: A549, derived from human lung carcinoma, and MCF-7, derived from human breast adenocarcinoma cells. These cell lines were procured from the Center for Stem Cell and Gene Therapies Research and Practice at Kocaeli University. The cells were cultured in Minimum Essential Medium (MEM; Thermo Fisher Scientific, Catalog No. 11095072, Waltham, MA, USA) supplemented with 10% heat-inactivated fetal bovine serum (FBS; Gibco, Catalog No. 16000044, USA) to provide the essential growth elements. Additionally, 1% penicillin-streptomycin (Pen/Strep; Gibco, 10,000 U/mL, Catalog No. 15140122, USA) was added to the culture medium to prevent bacterial contamination. The cells were incubated at 37 °C in a humidified atmosphere containing 5% CO_2_ to ensure optimal growth conditions. The culture medium was refreshed every 48 h to maintain nutrient balance and prevent the accumulation of metabolic waste. Upon reaching approximately 70–80% confluence, the cells were enzymatically detached using 0.25% trypsin-EDTA (Capricorn Scientific, Catalog Number: TRY-3B, Ebsdorfergrund, Germany) and transferred to new flasks for expansion. Following irradiation, the cells were returned to standard culture conditions and maintained for an additional five days to facilitate recovery and allow for subsequent analysis of radiobiological responses, including proliferation, survival, and morphological changes. Based on data from experimental studies [26], Table 1 summarizes the radiobiological parameters of the cell lines under investigation. These parameters, derived from photon clonogenic survival data fitted with the linear-quadratic (LQ) model, were used to assess and compare radiosensitivity. Although absolute values may vary slightly between studies owing to differences in irradiation conditions and fitting methodologies, A549 cells generally exhibit greater radiosensitivity than MCF-7 cells under conventional fractionation, as evidenced by their lower survival fraction at 2 Gy. Conversely, MCF-7 cells exhibited a lower α/β ratio, indicating heightened sensitivity to fraction size and a greater relative advantage of hypofractionated schedules. Both cell lines possessed α/β ratios within the moderately high range typical of tumor tissue. The “Radiosensitivity interpretation” column correlates each parameter set with its biological implication in the situation of fractionation sensitivity. The α/β ratio reflects the fraction-size sensitivity rather than the baseline radiosensitivity. A high α/β ratio (A549) suggests reduced reliance on fraction size, whereas a low α/β ratio (MCF-7) indicates a greater response to larger fraction doses. Therefore, the absolute radiosensitivity at conventional doses is more accurately reflected by the SF_2_ and D_10_ values, and the literature-based values for these parameters have been incorporated into the table.

### 2.2. Experimental Design, Treatment Planning, and Irradiation of the Cells

The in vitro irradiation setup was meticulously designed using a custom-built phantom filled with rice to simulate tissue-equivalent conditions in the human body. Cell culture flasks containing a monolayer (~1 mm) of adherent cells were strategically positioned in the phantom. A computed tomography (CT) scan of the configuration was acquired with a slice thickness of 1.5 mm to ensure precise treatment planning. Volumetric modulated arc therapy (VMAT) plans were generated using the Eclipse Treatment Planning System (v11.0; Varian Medical Systems, Palo Alto, CA, USA) with 6 MV photon beams in both the flattened filter (FF) and flattening filter-free (FFF) modes, employing single full arcs. Irradiation was conducted using a Varian Trilogy linear accelerator (Varian Medical Systems, Palo Alto, CA, USA) to examine the radiobiological effects on A549 human lung cancer and MCF-7 human breast adenocarcinoma cells, focusing on varying dose rates, dose per pulse, and beam-on time reduction across three single-fraction regimens (5, 10, and 20 Gy) using FF and FFF beams with 6 and 10 MV photon beams. Control flasks for each experimental set were maintained under identical environmental conditions but were not irradiated. All irradiation procedures were performed in triplicate to account for variability due to potential contamination and inconsistencies in plating efficiency, thereby ensuring reproducibility through standard deviation analysis of survival fractions. Owing to the differing plating efficiencies among the experimental groups, post-irradiation cell survival was assessed using the calculated survival fraction rather than direct viable cell counts. To standardize the comparisons between each experimental set, the survival fractions were normalized relative to their respective control groups. The rice-filled phantom was used as a reproducible tissue-equivalent irradiation medium to maintain comparable scatter conditions and geometric consistency across all VMAT delivery configurations during comparative in vitro irradiation experiments.

The Gross Tumor Volume (GTV) was delineated using computed tomography (CT) slices of a phantom, which included the outline of a flask filled with a 1 mm cell layer solution. Subsequently, the Planning Target Volume (PTV) was established by adding a 2 mm margin to the GTV to ensure comprehensive coverage of the flask (GTV) by 100% of the prescribed dose, mitigating potential setup errors during irradiation. Volumetric Modulated Arc Therapy (VMAT) plans were devised using a single arc to deliver 5, 10, and 20 Gy per fraction. The experimental setup and methodological specifications were defined and implemented in accordance with the procedures detailed in our previous study, thereby ensuring the consistency and comparability of the present work with our earlier findings [4]. The radiobiological effects of FFF beams were examined using two distinct experimental setups at single-fraction doses of 5, 10, and 20 Gy, designed to comparatively examine delivery-dependent parameters in vitro—including average dose rate (ADR), dose-per-pulse (DPP), and instantaneous dose rate (IDR)—under different clinically relevant beam configurations using single-arc VMAT. DPP was increased by using FFF beams and, where indicated, by raising the energy from 6FFF to 10FFF at a matched ADR. The IDR increased by shortening the source-to-surface distance (SSD) for the same phantom setup, changing only the flask position. The experiments were divided into two settings: Setting I (SSD = 90 cm) investigated the effects of ADR and DPP at 6 MV FF and 6 MV FFF (600 MU·min^−1^), as well as at increased ADR with 6 MV FFF (1400 MU·min^−1^) and 10 MV FFF (2400 MU·min^−1^). In Setting II, only the SSD was changed; the energy, nominal ADR (MU·min^−1^), field size, arc count, and delivery technique remained unchanged. Setting II (SSD = 75 cm) repeated the comparisons at a shorter SSD to elevate the IDR while maintaining the same nominal delivery rate conditions (6 MV FF and 6 MV FFF at 600 MU·min^−1^). The 10 MV FFF/2400 MU·min^−1^ condition (F) was evaluated exclusively within Setting I (SSD = 90 cm) as the high-dose-rate/high-DPP comparator. In each matched comparison (e.g., FF vs. FFF at the same ADR), plans utilized a single arc and were created to provide the same prescribed dose, with Monitor Units calculated by the Treatment Planning System (TPS) for each plan. The reference DPPs at the monitor chamber were ~0.28, 0.78, and 1.31 mGy/pulse for 6 MV FF, 6 MV FFF, and 10 MV FFF, respectively. At SSD = 75 cm, the DPP at the sample was considered ~44% higher than that at SSD = 90 cm, at approximately 0.40 mGy/pulse for 6 MV FF and 1.12 mGy/pulse for 6 MV FFF, consistent with the ~44% increase expected from inverse-square scaling relative to SSD = 90 cm. Table 2 provides a summary of all the experimental sessions. Quality control of all plans was assessed using an array dosimetry system by comparing the cumulative results of the delivered plans with the dose distribution of the calculated plans, based on the percentage passing-rate criterion of a gamma value of 3%/2 mm. Additionally, the output of the linear accelerator for each photon energy at a nominal dose rate of 600 MU/min was verified prior to cell irradiation using an ion chamber, in accordance with TRS-398 [31]. It should be noted, however, that the experimental design comparatively examined clinically relevant FFF and FF delivery configurations rather than fully isolating ADR, DPP, and IDR as physically independent variables, since several comparisons inherently involved simultaneous variation of beam energy, SSD, beam profile, and associated scatter conditions; the corresponding biological responses are therefore interpreted as associated with specific delivery configurations rather than as definitive isolated effects of individual physical parameters. Although the pretreatment quality assurance described above ensured that all delivered plans met institutional acceptance thresholds, direct sample-plane dosimetric verification at the cell-monolayer level was not performed for every delivery configuration, and minor residual dosimetric differences between configurations cannot be fully excluded. The rice-filled phantom likewise served as a reproducible, geometrically consistent comparative irradiation medium rather than as a fully water-equivalent reference; small dosimetric uncertainties inherent to such phantom-based in vitro setups are acknowledged as a contribution to the overall biological variability observed.

### 2.3. Quantification of Cell Viability After Radiotherapy

The assessment of cell viability of irradiated A549 human lung carcinoma and MCF-7 human breast adenocarcinoma cells was conducted using a water-soluble tetrazolium salt (WST-1) reagent (2-(4-iodophenyl)-3-(4-nitrophenyl)-5-(2,4-disulfophenyl)-2H-tetrazolium salt; Catalog No. 501594400, Roche Diagnostics, Laval, QC, Canada) through a colorimetric assay. This assay serves as an indirect measure of the viable cell count by evaluating metabolic activity. After irradiation, the cells underwent a 5-day incubation period under standard conditions (37 °C, 5% CO_2_). Subsequently, the culture medium in each well was carefully removed and replaced with MEM basal medium (Thermo Fisher) containing 10% WST-1 (Roche). The cells were then incubated for an additional 4 h to enable the cleavage of the tetrazolium salt by mitochondrial dehydrogenases in metabolically active cells. Absorbance was measured at 490 nm using a microplate spectrophotometer (VersaMax, Molecular Devices, San Jose, CA, USA). Each experimental condition was performed in triplicate to ensure reproducibility and statistical reliability. Blank wells containing only MEM basal medium (Thermo Fisher) and 10% WST-1 (Roche) without cells were included to account for background absorbance. All data were normalized to the absorbance values of the corresponding control (non-irradiated) cell populations. The optical density values obtained were used to determine relative cell viability and analyze the cytotoxic effects of different irradiation parameters.

### 2.4. Assessment of Colony Formation Capacity of Cells After Radiotherapy

To assess the clonogenic survival and proliferative capacity of irradiated cancer cells, a colony-forming unit (CFU) assay was performed. Post-irradiation under the specified experimental conditions, A549 (human lung carcinoma) and MCF-7 (human breast adenocarcinoma) cells were trypsinized, counted, and seeded at a density of 1000 cells per well into 6-well tissue culture plates containing complete growth medium. The cells were allowed to attach and proliferate under standard incubation conditions (37 °C, 5% CO_2_) for the initial 24-h period. Subsequently, the cultures were maintained for 14 days to facilitate colony development, with medium changes every 3–4 d. At the end of the incubation period, the colonies were gently washed with phosphate-buffered saline (PBS; Thermo Fisher Scientific, Catalog Number: 10010023, Waltham, MA, USA), fixed with 100% methanol (Thermo Fisher Scientific, Catalog Number: 176840010, Waltham, MA, USA) for 15 min, and stained with a 0.5% crystal violet solution (Thermo Fisher Scientific, Catalog Number: 405835000, Waltham, MA, USA) for 30 min at room temperature. Excess dye was removed by rinsing with distilled water, and the plates were air-dried. Colonies comprising ≥50 cells were manually counted under an inverted microscope, and the colony formation rate was calculated as a percentage relative to the control (non-irradiated) group. The assay was performed in triplicate for each experimental condition to ensure statistical reliability. This procedure facilitated the evaluation of the long-term survival and reproductive integrity of cells following exposure to various irradiation regimens.

### 2.5. Statistical Analyses

All experimental procedures, including WST-1 cell viability and CFU assays, were independently conducted in triplicate to ensure the reproducibility and reliability of the results. These assays were performed on both A549 human lung adenocarcinoma and MCF-7 human breast adenocarcinoma cell lines under varying dose rates, increased DPP, and irradiation conditions. Quantitative results were expressed as mean ± standard deviation (SD). Comparative statistical analyses between the experimental and control groups were conducted using two-tailed Welch *t*-tests for independent sample analyses. A *p*-value less than 0.05 (*p* < 0.05) was considered statistically significant. This significance threshold was applied to evaluate the radiobiological effects of different dose rates, dose per pulse (DPP), and IDR durations on cell viability and clonogenic potential. All statistical computations and data visualizations were performed using IBM SPSS Statistics for Windows (version 20.0; IBM Corp., Armonk, NY, USA). Data integrity was ensured by examining the consistency across replicates and assessing the distributional assumptions required for the parametric analysis. This statistical approach provided robust insights into the biological impact of high-dose radiotherapy delivered through flattening filter and flattening filter-free beam configurations. The correlation between WST and clonogenic outcomes was assessed using Kendall’s tau-b, computed within each single-dose level (5, 10, and 20 Gy) and restricted to FFF deliveries (B, C, E, and F) to focus on the effects of ADR/DPP/IDR. Primary analyses used delivery-relative changes (Δ vs. B: 6FFF/600, SSD = 90 cm) to comparatively assess treatment-configuration-associated responses (ADR: C; IDR: E; high-ADR/DPP: F). Two-sided exact/permutation *p*-values and bootstrap 95% confidence intervals were reported.

## 3. Results

**Figure 1:**
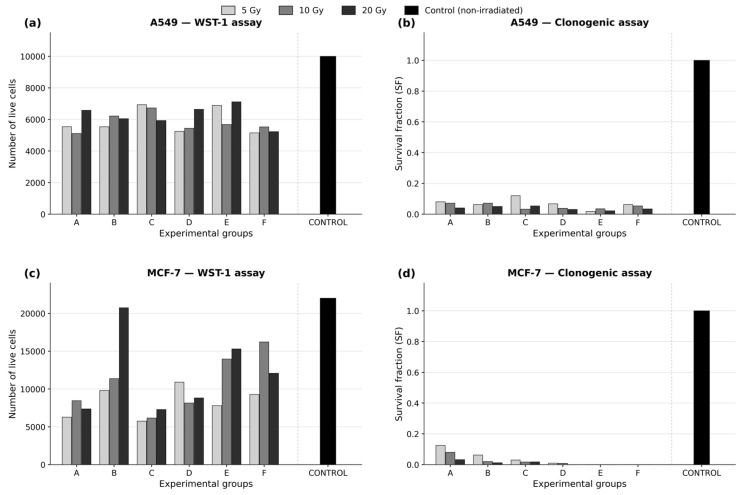
Radiobiological responses across delivery parameters and doses. (**a**) A549 WST-1 cell viability (% of control); (**b**) A549 clonogenic survival; (**c**) MCF-7 WST-1 cell viability; (**d**) MCF-7 clonogenic survival. Contrasts highlighted the doses from 5 Gy to 20 Gy in a single fraction, and each triple experiment group illustrates the result of dose rate (6FFF 1400 vs. 600 MU·min^−1^ at SSD = 90 cm and 10FFF/2400 vs. 6FFF/1400 at SSD = 75 cm), DPP (6FFF vs. 6FF at 600 MU·min^−1^, SSD = 90 cm), and IDR (SSD = 90 vs. 75 cm at 6FFF/600; B vs. E labels). The experiment group labels A–F correspond to Table 2.

The radiobiological impact of the delivery parameters associated with FFF beams was comprehensively investigated using two complementary experimental configurations, each specifically structured to comparatively evaluate the relative contributions of the ADR, DPP, and IDR under single-fraction exposures of 5, 10, and 20 Gy. In Setting I (SSD = 90 cm), variations in the ADR and DPP were systematically assessed using four distinct beam configurations: A (6 MV FF at 600 MU·min^−1^), B (6 MV FFF at 600 MU·min^−1^), C (6 MV FFF at 1400 MU·min^−1^), and F (10 MV FFF at 2400 MU·min^−1^). Within this framework, the 10 MV FFF beam at 2400 MU·min^−1^ functioned as the high-dose-rate reference, representing the clinically achievable maximum nominal rate and serving as a pragmatic comparator rather than an energy escalation experiment. In Setting II (SSD = 75 cm), the nominal delivery conditions of 600 MU·min^−1^ for both the 6 MV FF and 6 MV FFF beams were preserved, with identical geometry except for the reduced SSD. This geometric modification selectively enhanced the IDR at the cell plane, thereby enabling the evaluation of the IDR effects independent of the ADR (configurations D and E, respectively). Biological outcomes were quantified through WST-1 metabolic and clonogenic colony-formation assays, with the latter designated as the principal endpoint because of its established relevance for long-term proliferative potential. The results suggested that the delivery-dependent effects were cell line-specific and dose-dependent. A549 cells exhibited only modest sensitivity, with significant ADR-associated reductions in clonogenic survival observed primarily at 10 Gy, consistent with their higher α/β ratio (~12.4 Gy) and reduced fraction size sensitivity. In contrast, MCF-7 cells displayed more pronounced decrements in clonogenic survival at 5–10 Gy when exposed to higher ADR and DPP, aligning with their lower α/β ratio (~4.1 Gy) and heightened sensitivity to larger fraction sizes. At 20 Gy, inter-arm differences largely diminished in both cell lines, reflecting the near-saturation of lethal damage. Importantly, WST-1 results did not consistently parallel clonogenic outcomes, underscoring that metabolic viability does not necessarily reflect reproductive survival in cancer cells. Collectively, these findings indicate, as shown in Figure 1, that FFF beam delivery parameters-particularly ADR and DPP-exert nuanced, cell-type-specific radiobiological effects, with implications for the optimization of hypofractionated regimens in clinical practice.

### 3.1. Effects of Gradual Dose Rate Elevation on Tumor Cell Survival

**Figure 2:**
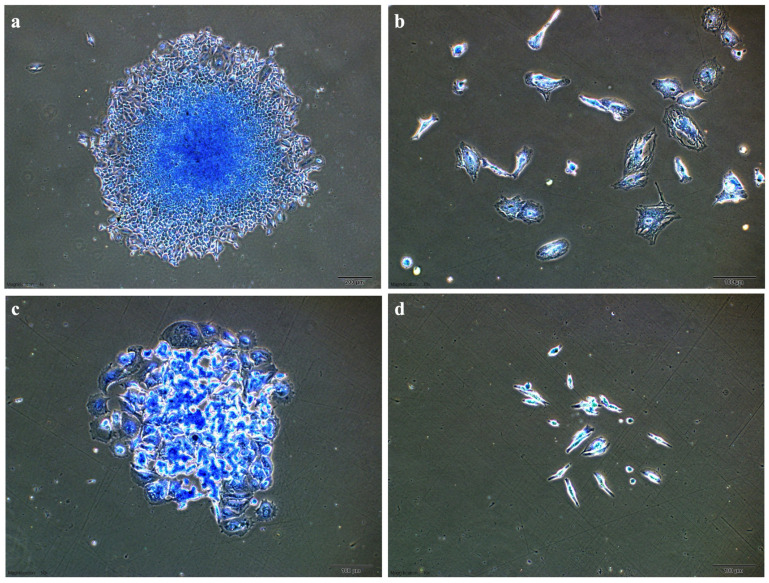
Representative plates derived from assay analysis demonstrating the impact of nominal dose rate (ADR) for A549 (**a**,**b**) and MCF-7 (**c**,**d**). (**a**) B2: 6 MV FFF, 600 MU·min^−1^, 10 Gy, SSD = 90 cm-many colonies remain, indicating ongoing proliferative capacity. (**b**) F3: 10 MV FFF, 2400 MU·min^−1^, 20 Gy, SSD = 90 cm-colonies are sparse/smaller, indicating marked suppression of proliferation. (**c**) B1: 6 MV FFF, 600 MU·min^−1^, 5 Gy, SSD = 90 cm-many colonies present. (**d**) C2: 6 MV FFF, 1400 MU·min^−1^, 10 Gy-decreased survival fraction. Scale bar: 100 and 200 µm.

In the geometric configuration of Setting I (SSD = 90 cm), four clinically relevant beam delivery conditions were evaluated—A (6 MV FF at 600 MU·min^−1^), B (6 MV FFF at 600 MU·min^−1^), C (6 MV FFF at 1400 MU·min^−1^), and F (10 MV FFF at 2400 MU·min^−1^)—at single-fraction doses of 5, 10, and 20 Gy. Condition F was analyzed only within Setting I and was not part of the SSD-modified Setting II comparisons. A dose-dependent reduction in survival fractions (SFs) across all conditions was indicated by clonogenic survival analysis of A549 cells. The raw colony-forming values were recorded as follows: at 5 Gy, SFs of 0.0725 (A), 0.0625 (B), 0.205 (C), and 0.0755 (F); at 10 Gy, 0.067 (A), 0.0765 (B), 0.0305 (C), and 0.049 (F); and at 20 Gy, 0.0535 (A), 0.042 (B), 0.059 (C), and 0.031 (F). Statistical analyses using Welch’s *t*-test supported that at 5 Gy, none of the pairwise comparisons reached significance (all *p* > 0.05), although non-significant trends toward reduced SFs at higher dose rates were noted (e.g., A vs. B *p* = 0.095; B vs. C *p* = 0.073). At 10 Gy, a significant difference was observed between A (6 MV FF at 600 MU·min^−1^) and C (6 MV FFF at 1400 MU·min^−1^) (*p* = 0.044), with lower SFs recorded at the elevated nominal ADR. Other contrasts at this dose, including B vs. C (*p* = 0.060), B vs. F (*p* = 0.065), and A vs. F (*p* = 0.086), did not achieve statistical significance but trended in the same direction, indicating reduced survival under higher ADR/DPP ratios. However, at 20 Gy, all comparisons were non-significant (e.g., C vs. F *p* = 0.087), consistent with the saturation of lethal damage at this high dose. Taken together, these results indicated that a measurable ADR effect was exhibited by A549 cells, centered around the intermediate dose of 10 Gy—particularly between 6FFF at 1400 MU·min^−1^ and 6FF at 600 MU·min^−1^—while at lower and higher doses, the effect was diminished or absent. Increased cell death was therefore observed at 5–10 Gy under higher nominal ADR, whereas at 20 Gy, the effect was attenuated, and damage appeared closer to saturation. This pattern was consistent with the higher α/β ratio of A549 (~12.4 Gy), which implied an overall limited fraction-size sensitivity. Consequently, in single-fraction tests, the incremental benefit of a higher ADR was modest and diminished at very large doses. Representative clonogenic plates for A549 and MCF-7 cells are presented in Figure 2. Although the doses differed between the panels, the images illustrated the qualitative direction of the ADR effects. Numerous surviving colonies were visible under B1 (6FFF at 600 MU·min^−1^, 5 Gy), indicating that a substantial fraction of irradiated A549 cells retained their proliferative capacity at this nominal dose rate (Figure 2a). In contrast, a marked reduction in both colony number and size was observed under F3 (10 MV FFF at 2400 MU·min^−1^, 20 Gy), consistent with substantial suppression of post-irradiation proliferative capacity under conditions of elevated dose rate combined with a large single-fraction exposure (Figure 2b). For MCF-7 cells, visible colony formation was detected at the lower ADR of 6 MV FFF at 600 MU·min^−1^ and 5 Gy, reflecting relatively preserved survival and reproductive potential (Figure 2c). However, under C2 (6 MV FFF at 1400 MU·min^−1^, 10 Gy), both colony number and size were significantly reduced compared with the corresponding lower-dose panel, underscoring the heightened sensitivity of MCF-7 cells to increased nominal ADR in conjunction with larger single-fraction doses, consistent with the more pronounced radiobiological effects observed in this cell line under these conditions (Figure 2d).

At an SSD of 90 cm, viable cell counts in the A549 cell line were evaluated using the WST-1 cell viability assay under four beam delivery conditions: 600 MU·min^−1^ (6 MV FF), 600 MU·min^−1^ (6 MV FFF), 1400 MU·min^−1^ (6 MV FFF), and 2400 MU·min^−1^ (10 MV FFF). The corresponding per-dose mean values (A/B/C/F) were 5292.7, 5779.9, 7246.5, and 5148.3 at 5 Gy; 5199.9, 5880.0, 7447.3, and 5619.9 at 10 Gy; and 7823.6, 5948.5, 6082.8, and 5390.2 at 20 Gy. Statistical analysis with Welch’s two-sample tests indicated that only one contrast for the ADR reached significance: at 5 Gy, condition A (6FF/600 MU·min^−1^) versus condition C (6FFF/1400 MU·min^−1^) produced a significant difference (*p* = 0.043). All other pairwise comparisons at 5 Gy remained non-significant (A vs. B, *p* = 0.293; B vs. C, *p* = 0.072; B vs. F, *p* = 0.232; A vs. F, *p* = 0.663; and C vs. F, *p* = 0.094). At 10 Gy, none of the contrasts achieved significance (A vs. B, *p* = 0.275; A vs. C, *p* = 0.192; A vs. F, *p* = 0.087; B vs. C, *p* = 0.237; C vs. F, *p* = 0.234; B vs. F, *p* = 0.578), and at 20 Gy all comparisons likewise remained non-significant (A vs. B, *p* = 0.372; A vs. C, *p* = 0.393; A vs. F, *p* = 0.298; B vs. C, *p* = 0.542; C vs. F, *p* = 0.087; B vs. F, *p* = 0.119). Overall, the WST-1 assay findings suggested that the ADR effect in A549 cells was limited, with only a single significant difference observed at 5 Gy (A vs. C) and no notable differences at 10 or 20 Gy doses. When interpreted in the elevated α/β ratio of this line (~12.4 Gy), these results indicated a reduced sensitivity to fraction size and only a modest incremental contribution of delivery rate under single-fraction conditions. Consequently, ADR-related alterations in metabolic viability appeared minimal in A549 cells and diminished further at higher single-dose treatment (Figure 2).

Clonogenic survival analyses of MCF-7 cells across the ADR arms (settings A, B, C, and F) were conducted under beam conditions of 600 MU·min^−1^ (6 MV FF), 600 MU·min^−1^ (6 MV FFF), 1400 MU·min^−1^ (6 MV FFF), and 2400 MU·min^−1^ (10 MV FFF), respectively, at an SSD of 90 cm. The resulting survival fractions were 0.1295, 0.0800, 0.1540, and 0.0015 at 5 Gy; 0.0880, 0.0325, 0.0130, and 0.00225 at 10 Gy; and 0.0390, 0.0155, 0.0230, and 0.0015 at 20 Gy. Concurrently, the WST-1 cell viability assay, performed under identical SSD conditions, yielded live cell counts, expressed as a fraction of control, of 6584.4, 11,001.2, 5320.5, and 8724.6 at 5 Gy; 10,183.0, 14,090.9, 5851.6, and 12,470.3 at 10 Gy; and 7612.5, 17,519.5, 6736.5, and 11,570.1 at 20 Gy, for A, B, C, and F, in turn. Welch’s two-sample tests indicated ADR-related differences: at 5 Gy, the comparison between C and F reached significance (*p* = 0.045), whereas other contrasts were not significant (A vs. B *p* = 0.149; A vs. C *p* = 0.154; A vs. F *p* = 0.108; B vs. C *p* = 0.100; B vs. F *p* = 0.273). At 10 Gy, no significant differences were identified (all *p* > 0.05), and at 20 Gy, significant differences were detected for A vs. F (*p* = 0.043) and C vs. F (*p* = 0.024), while all other contrasts remained non-significant (A vs. B *p* = 0.198; A vs. C *p* = 0.332; B vs. C *p* = 0.176; B vs. F *p* = 0.309). Collectively, the WST-1 results revealed discernible ADR effects, predominantly when compared with the high-dose-rate arm (F), with significant differences observed at 5 and 20 Gy, but not at 10 Gy. These findings, when interpreted in light of the lower α/β ratio of MCF-7 (~4.1 Gy), indicated a heightened sensitivity to larger single-fraction exposures. However, as the WST-1 assay measures metabolic viability, these observations were considered supportive, yet secondary, to clonogenic survival outcomes. Within-FFF beam contrasts, comparing condition B (600 MU·min^−1^) with condition C (1400 MU·min^−1^), were not statistically significant at 5, 10, or 20 Gy in the WST-1 data (Welch *p* = 0.655/0.346/0.356). In contrast, clonogenic survival analysis of the same comparison (B vs. C) demonstrated significantly reduced survival at 10 Gy (*p* = 0.032; Figure 2d), whereas no differences were observed at 5 or 20 Gy. Comparisons with the high-rate comparator (A vs. F: 10FFF at 2400 MU·min^−1^) showed that clonogenic survival was significantly reduced at both 5 Gy (*p* = 0.012; decreasing from 0.1295 to 0.0015; Figure 2c) and 10 Gy (*p* = 0.044; decreasing from 0.0880 to 0.00225; Figure 2d), whereas no significant difference was observed at 20 Gy (*p* = 0.105). Overall, MCF-7 cells exhibited larger decrements in clonogenic survival under higher nominal dose rates, most evident at 5–10 Gy, whereas the effects at 20 Gy were less consistently supported by statistical significance. This pattern was consistent with the lower α/β ratio of MCF-7 (~4.1 Gy), reflecting greater sensitivity to large single-fraction exposures and highlighting that the ADR-associated enhancement of effect was most apparent within the intermediate-to-large dose range.

### 3.2. Radiobiological Effects of DPP- and IDR-Associated Reduction in Clonogenic Survival

To investigate the influence of DPP on A549 cells at a constant average dose rate, two experimental contrasts were examined: 6 MV FF (A: 600 MU·min^−1^) versus 6 MV FFF (B: 600 MU·min^−1^) at SSD = 90 cm, representing an increase in DPP from approximately 0.28 to 0.78 mGy/pulse; and within FFF beams, comparison of D (600 MU·min^−1^) and E (600 MU·min^−1^) at SSD = 75 cm, corresponding to an increase in DPP from approximately 0.40 to 1.12 mGy/pulse. Under the matched ADR condition (600 MU·min^−1^, SSD = 90 cm), no statistically significant differences were detected in clonogenic survival between A and B, with survival fractions changing from 0.0725 to 0.0625 at 5 Gy (*p* = 0.095), from 0.060 to 0.0765 at 10 Gy (*p* = 0.308), and from 0.0535 to 0.042 at 20 Gy (*p* = 0.555). Similarly, WST-1 cell viability assay values did not differ significantly at 5, 10, or 20 Gy (*p* = 0.293, 0.275, and 0.373, respectively). When the SSD was shortened to 75 cm to elevate both DPP and IDR, clonogenic survival decreased consistently in FFF (E) compared with FF (D), although these differences did not reach statistical significance (*p* = 0.097, 0.193, and 0.112 at 5, 10, and 20 Gy, respectively). Interestingly, the WST-1 cell viability assay revealed a paradoxical increase in viable cell count for E at 5 Gy (*p* = 0.0176), whereas no differences were observed at 10 or 20 Gy (*p* = 0.542, 0.441). Taken together, these findings suggest that under conditions of elevated DPP and IDR, A549 cells demonstrated clonogenic suppression with FFF delivery, whereas the WST-1 cell viability assay did not parallel this pattern and, at 5 Gy, even suggested preserved metabolic activity despite reduced reproductive survival (Figure 2a,b).

In MCF-7 cells, increasing DPP under matched ADR conditions consistently reduced clonogenic survival, with the strongest effect at 10 Gy, where survival decreased from 0.0880 to 0.0325 (*p* = 0.082, near-significant), while differences at 5 and 20 Gy were non-significant (*p* = 0.197 and 0.136, respectively). The WST-1 cell viability assay values for these conditions did not show significant alterations (*p* = 0.149, 0.367, and 0.198 at 5, 10, and 20 Gy, respectively). When the SSD was reduced to 75 cm (D vs. E), thereby simultaneously increasing the DPP and IDR, clonogenic assays revealed stronger cytotoxic effects with FFF, particularly at 10 Gy (*p* = 0.064, near-significant), whereas, at 20 Gy, a non-significant reversal was observed (*p* = 0.548). The WST-1 cell viability assay suggested a dose-dependent trend in the opposite direction, with FFF showing reduced efficacy at 5 Gy but relatively greater suppression at 10 and 20 Gy, although none of these differences achieved statistical significance (*p* = 0.094, 0.107, 0.064, respectively). These observations underscored that, in MCF-7 cells, characterized by a lower α/β ratio (~4.1 Gy), elevated DPP and IDR were associated with greater reductions in clonogenic survival at intermediate doses, most notably at 10 Gy, while the WST-1 cell viability assay failed to consistently capture these differences (Figure 2c,d).

### 3.3. Correlation Between WST-1 Cell Viability and Clonogenic Survival Outcomes

Correlation analyses were conducted to investigate the association between WST-1 cell viability assay results and clonogenic survival fractions, categorized by cell line and specifically linked to the FFF delivery settings designed to explore variations in ADR, DPP, and IDR at single-fraction exposures of 5, 10, and 20 Gy. For each dose, delivery-related changes were calculated relative to the baseline FFF condition (B: 6 MV FFF at 600 MU·min^−1^, SSD = 90 cm). These changes were represented as ΔWST-1 = WST-1_arm_ − WST-1_B_ and ΔSF = SF_arm_ − SF_B_ for three conditions: condition C, indicating an increase in the nominal ADR (6 MV FFF at 1400 MU·min^−1^, SSD = 90 cm); condition E, indicating an increase in the IDR at the sample plane (6 MV FFF at 600 MU·min^−1^, SSD = 75 cm); and condition F, indicating a combined increase in the nominal ADR and DPP (10 MV FFF at 2400 MU·min^−1^, SSD = 90 cm). Correlations between ΔWST-1 and ΔSF were subsequently computed using Kendall’s τ-b with two-sided exact permutation *p*-values and bootstrap 95% confidence intervals (CIs).

In A549 cells, comparisons of delivery conditions relative to the FFF baseline (Δ vs. B: 6 MV FFF at 600 MU·min^−1^, SSD = 90 cm) consistently revealed negative, albeit non-significant, correlations between the WST-1 cell viability assay and clonogenic survival, with the most pronounced effect observed at 5 Gy. Specifically, Kendall’s τ_b_ values were −1.00 at 5 Gy (*p* = 0.333, 95% CI −1.00 to −1.00), −0.33 at 10 Gy (*p* = 1.000, 95% CI −1.00 to 1.00), and −1.00 at 20 Gy (*p* = 0.333, 95% CI −1.00 to −1.00), based on three delivery contrasts per dose: condition C, indicating an increase in ADR (6 MV FFF at 1400 MU·min^−1^, SSD = 90 cm); condition E, indicating an increase in IDR achieved by reducing the SSD (6 MV FFF at 600 MU·min^−1^, SSD = 75 cm); and condition F, indicating a combined increase in nominal ADR and DPP (10 MV FFF at 2400 MU·min^−1^, SSD = 90 cm). Although none of these correlations achieved statistical significance (*p* < 0.05), the direction of the effect suggested that increases in ADR and, more notably, increases in IDR were associated with reduced clonogenic survival, particularly at 5–10 Gy, without a corresponding decrease in WST-1 cell viability. The high-rate comparator (condition F) exhibited a similar pattern, where clonogenic survival did not improve, but the WST-1 assay values remained stable. Consequently, the overall rank association remained negative (Figure 2a,b). These findings implied that in A549 cells, increases in ADR and IDR that enhance clonogenic cell death were not reflected by the WST-1 assay in a monotonic manner, consistent with the interpretation that the WST-1 assay measures metabolic activity in surviving cells rather than their long-term colony-forming potential. This divergence aligned with the higher α/β ratio of A549 (~12.4 Gy), where single-fraction doses exceeding approximately 10 Gy tend to saturate clonogenic endpoints, whereas metabolic activity measured by the WST-1 assay remains relatively unaffected.

In MCF-7 cells, when comparing delivery-related contrasts to the FFF baseline (Δ vs. B: 6 MV FFF at 600 MU·min^−1^, SSD = 90 cm), a consistent pattern of negative correlations emerged between clonogenic survival and the WST-1 cell viability assay. The most significant inverse correlation was observed at 10 Gy, which aligned with the dose level where adjustments in ADR and IDR most effectively reduced clonogenic survival. Specifically, Kendall’s τ_b_ values were −0.33 at 5 Gy (*p* = 1.000, 95% CI −1.00 to 1.00), −1.00 at 10 Gy (*p* = 0.333, 95% CI −1.00 to −1.00), and −0.33 at 20 Gy (*p* = 1.000, 95% CI −1.00 to 1.00), based on three delivery contrasts per dose: condition C, indicating an increase in ADR (6 MV FFF at 1400 MU·min^−1^, SSD = 90 cm); condition E, indicating an increase in IDR by reducing the SSD (6 MV FFF at 600 MU·min^−1^, SSD = 75 cm); and condition F, indicating a combined increase in nominal ADR and DPP (10 MV FFF at 2400 MU·min^−1^, SSD = 90 cm). Mechanistically, increases in ADR (B vs. C) and particularly in IDR (B vs. E) led to notable reductions in clonogenic survival at 5–10 Gy; however, the WST-1 cell viability assay did not show a corresponding decrease, resulting in consistently negative rank associations (Figure 2c,d). The high-rate comparator (condition F) followed a similar trend, showing significant clonogenic depletion without a corresponding reduction in the metabolic viability. Although none of the correlations reached statistical significance (*p* < 0.05) due to the limited number of contrasts (*n* = 3 per dose), the consistent direction of effect suggested that substantial decreases in clonogenic survival were not matched by proportional declines in the WST-1 outcomes. Overall, in MCF-7 cells, consistent with their lower α/β ratio (~4.1 Gy) and increased sensitivity to large single-fraction exposures, delivery parameters such as increased ADR and IDR tended to reduce clonogenic survival at 5–10 Gy. However, the WST-1 cell viability assay did not capture this suppression in a monotonic manner, underscoring the distinct biological dimensions measured by metabolic viability versus reproductive capacity.

## 4. Discussion

The clinical implementation of FFF radiotherapy was regarded as controversial, since evidence regarding its efficacy and safety was reported to differ across several preclinical investigations. In several in vitro studies, no statistically significant differences in cell survival were observed between healthy and malignant cell populations irradiated with conventional dose rates and those exposed to high-dose-rate FFF beams [9,21,32]. However, other reports have suggested a therapeutic advantage for high-dose-rate FFF radiotherapy [10,33]. In particular, irradiation under high-dose-rate FFF conditions was associated with enhanced efficacy of tumor-cell killing, while radiation-induced damage in surrounding normal tissues was simultaneously reduced. Through these observations, the potential role of high-dose-rate FFF irradiation in improving the therapeutic ratio was emphasized. In light of the divergent findings reported across preclinical studies, further experimental evaluation was required to clarify the biological implications of FFF irradiation delivered under varying beam characteristics. The present analysis suggested that delivery configurations associated with variations in ADR, DPP, and IDR may differentially influence tumor cell response, thereby underscoring the complexity of the radiobiological effects associated with high-dose-rate FFF beams. By systematically examining both short-term metabolic activity through the WST-1 cell viability assay and long-term clonogenic survival via the colony formation assay, distinct insights were obtained into the temporal dynamics of radiation-induced cytotoxicity in tumor cell lines with different radiosensitivity profiles. Our findings highlighted that variations in ADR, DPP, and IDR were not uniformly translated into detectable differences across all cellular assays, suggesting that the biological impact of FFF irradiation may be dependent on the assay used. While the WST-1 assay provided valuable information on acute metabolic activity, clonogenic survival was revealed to be more sensitive in capturing long-term proliferative capacity, which is particularly relevant for assessing tumor control probability. Furthermore, by interpreting these results in the context of α/β ratios and intrinsic radiosensitivity, we differentiated the delivery effects attributable to distinct beam parameters, particularly under single-fraction exposures of 5, 10, and 20 Gy. Taken together, these observations emphasized that the radiobiological outcomes of FFF irradiation likely reflect the combined influence of multiple delivery-related parameters but must also be considered in relation to the interplay between beam characteristics, cellular radiosensitivity, and the biological endpoint under investigation. This perspective may explain the apparent discrepancies in the literature, where some studies failed to detect significant differences between conventional and high-dose-rate irradiation, whereas others suggested a therapeutic advantage of FFF beams in enhancing tumor-cell kill while sparing normal tissues [34]. Ultimately, the results of the current investigation contributed to a more nuanced understanding of how dose-rate-related parameters influence cellular responses and may inform future optimization strategies for the clinical application of FFF radiotherapy.

Upon comparing the irradiated groups with the non-irradiated controls, it became evident that there was a notable decline in cell viability across most experimental conditions. This observation supported that the radiation doses administered had cytotoxic effects on both A549 and MCF-7 cells (Figure 1a,c). This decline was not observed uniformly, as certain delivery settings produced the most pronounced divergence, whereas others did not result in statistically significant reductions in viability. In particular, the WST-1 cell viability assay did not consistently demonstrate a measurable decrease in absorbance values, indicating that under specific conditions, the radiation dose was insufficient to significantly suppress metabolic activity. Nevertheless, a complete absence of colony formation was revealed by the clonogenic assay under these same conditions (Figure 1b,d), thereby highlighting the distinction between short-term metabolic viability and long-term proliferative potential. On this basis, clonogenic survival was reaffirmed as the gold standard endpoint for assessing radiotherapy efficacy, since cells that retained metabolic activity were no longer capable of sustaining indefinite proliferation. Within the framework of Setting I (SSD = 90 cm), four clinically relevant beam configurations spanning the therapeutic dose-rate range were evaluated: A (6 MV FF/600 MU·min^−1^), B (6 MV FFF/600 MU·min^−1^), C (6 MV FFF/1400 MU·min^−1^), and F (10 MV FFF/2400 MU·min^−1^). When the outcomes were interpreted in light of radiobiological modeling, the differences between A549 (α/β ≈ 12.4 Gy) and MCF-7 (α/β ≈ 4.1 Gy) cells were attributed to their distinct radiosensitivity profiles [27,28,29,30]. In A549 cells, which exhibited a higher α/β ratio, limited fraction-size sensitivity and modest ADR-related effects were detected, most evident at 10 Gy, where clonogenic suppression was significant (Figure 1b). In contrast, MCF-7 cells, characterized by a lower α/β ratio, indicated greater sensitivity to large single-fraction doses, with statistically significant decrements in clonogenic survival observed following high-dose-rate 10 MV FFF irradiation (Figure 1d). These observations indicated that ADR- and DPP-associated effects were highly cell line-dependent and were most evident in the intermediate-dose range (5–10 Gy), where the clonogenic potential was most vulnerable to accelerated delivery. Overall, the results of our study strengthened the concept that while metabolic assays, such as the WST-1 cell viability assay, are beneficial for understanding radiobiological effects, they were not exhaustive. These assays focused on short-term mitochondrial activity rather than the long-term aspects of reproductive integrity. The divergence between WST-1 and clonogenic findings (Figure 1a–d) was consistent with prior reports and further supported the view that clonogenic endpoints remain superior for predicting cell survival and tumor control probability. Moreover, the heterogeneity observed between A549 and MCF-7 responses was interpreted as reflecting the influence of intrinsic radiosensitivity and α/β phenotype on the biological consequences of high dose-rate FFF irradiation. Variability in study findings may contribute to the discrepancies noted in the literature, with some studies indicating minimal differences between conventional and FFF beams, while others highlighted potential therapeutic benefits linked to high-dose rate delivery [35]. Based on this, the results of the current study were deemed to offer additional proof that the advantages of FFF beam delivery depend on the condition and were likely to be most significant in tumor types with low α/β ratios and heightened sensitivity to hypofractionated treatments.

In the A549 lung carcinoma cell line, a discernible sensitivity to ADR was identified within the clinically relevant FFF range. Specifically, comparisons between the flattened 6 MV beam at 600 MU·min^−1^ (A) and the FFF beam at 1400 MU·min^−1^ (C) indicated reduced cell viability at the higher ADR at 5 Gy (WST assay, *p* = 0.043) and significantly diminished clonogenic survival at 10 Gy (*p* = 0.044). Within the FFF delivery conditions, the comparison between 6 MV FFF at 600 MU·min^−1^ (B) and 1400 MU·min^−1^ (C) showed a non-significant yet directionally consistent reduction in survival fraction at 10 Gy (*p* = 0.060). In contrast, comparisons with the high-dose-rate 10 MV FFF condition at 2400 MU·min^−1^ (F) were largely non-significant, although a modest trend was observed in the WST assay at 10 Gy (*p* ≈ 0.087). Collectively, these results aligned with the established high α/β ratio of A549 (~12.4 Gy), suggesting limited sensitivity to fraction size. Consequently, the most pronounced ADR-associated effect was confined to the intermediate-dose range (≈10 Gy), where clonogenic suppression was detectable without changes in the fraction size. These findings are consistent with and complement the results of Nakano et al. [20], who reported that high-dose-rate FFF photons neither altered the survival of A549 cells nor produced significant effects under single-fraction exposures ≤ 8 Gy. Thus, in A549 cells, ADR sensitivity appears subtle, state-dependent, and most evident within a restricted dose window, diminishing at both lower and higher single-fraction doses. Conversely, MCF-7 breast adenocarcinoma cells exhibited a different pattern. The ADR-only changes within the FFF range (B vs. C) were not statistically significant; however, reductions in survival became apparent when compared with the high-dose-rate 10 MV FFF beam. Specifically, clonogenic survival was significantly lower at 5 Gy (*p* = 0.012) and 10 Gy (*p* = 0.044) in the A vs. F comparison, while WST analysis revealed a significant decrease at 20 Gy (*p* = 0.045). Additional WST-based reductions were detected when comparing C vs. F at both 5 Gy (*p* = 0.045) and 20 Gy (*p* = 0.024). These observations are consistent with the lower α/β ratio of MCF-7 (~4.1 Gy), indicating increased sensitivity to large single fractions and a phenotype that is more susceptible to accelerated delivery. The results suggest that high nominal dose rates and elevated dose-per-pulse delivery amplify cytotoxicity in the large-dose regime, consistent with the biological expectation of an enhanced effect in low α/β systems. Furthermore, our findings align with those of others who demonstrated delivery-related variations in MCF-7 xenografts at the level of immunohistochemical markers (e.g., proliferative and oxidative stress indicators) and corroborated these findings with transcriptomic data showing altered ER-associated degradation pathways and glutamatergic signaling under FFF versus FF irradiation conditions [36]. Taken together, the two cell lines exhibited distinct dose-rate response profiles that reflected their intrinsic radiobiological characteristics. A549 cells, characterized by a higher α/β ratio, displayed selective ADR sensitivity at approximately 10 Gy but little or no effect at other dose levels, whereas MCF-7 cells, with a lower α/β ratio, showed more consistent and statistically significant decrements in survival as the single-fraction dose and nominal delivery rate increased. These results emphasized that the effects of ADR and delivery parameters are both dose- and phenotype-dependent, highlighting the importance of integrating α/β-based radiosensitivity into the interpretation of FFF beam radiobiology.

Across single-fraction exposures up to 20 Gy, the influence of ADR on the two cancer cell lines was evaluated; nevertheless, no biologically significant effects were identified overall. Within the limits imposed by the small per-arm sample size, the ADR effect was not statistically or biologically significant; however, it was stronger and more internally consistent in MCF-7 cells at larger single fractions, whereas A549 cells exhibited a selective ADR sensitivity centered around 10 Gy. These between-line differences were directionally consistent with the working α/β for each phenotype: A549 (high α/β) was relatively less influenced by fraction size yet still showed a detectable ADR susceptibility, while MCF-7 (low α/β) displayed greater decrements in metabolic viability and clonogenic survival as the single-fraction dose and nominal delivery rate increased [37,38]. When the SSD was reduced to 75 cm in Setting II, thereby increasing the DPP and IDR at the sample plane while holding MU·min^−1^ constant, the most consistent clonogenic suppression in both lines was produced at 5–10 Gy. In contrast, a pure DPP comparison at equivalent ADR (6FF vs. 6FFF at 600 MU·min^−1^) resulted in no alterations in survival. Increasing DPP produced no statistically significant change in clonogenic survival for A549 across 5–20 Gy, with a non-significant sparing effect at 5 Gy that dissipated at higher doses. In MCF-7 cells, higher DPP at the same ADR yielded a consistent reduction in the survival fraction, with the 10 Gy comparison approaching significance, an effect that aligned with the expectation that a lower α/β phenotype is more sensitive to large single fractions. These results were concordant with the accepted α/β values-approximately 12.4 Gy for A549 (less sensitive to fraction size) versus ~4.1 Gy for MCF-7 (more sensitive to larger fractions)-and with the general observation that effective α/β may vary by dose range and endpoint [17,29]. Overall, A549 exhibited modest delivery-rate sensitivity, with the clearest effects appearing in the lower-to-intermediate single-dose window and diminishing by 20 Gy, a pattern consistent with its higher α/β (~12.4 Gy) and correspondingly reduced fraction-size sensitivity. These findings were also concordant with reports emphasizing that removing the flattening filter increases DPP and enables higher nominal dose rates while shortening the beam-on time, thereby reducing intrafraction repair opportunities at non-FLASH rates [39,40]. Our findings suggest that DPP alone exerts limited influence on A549 but may accentuate killing in MCF-7 around intermediate hypofractionated doses (≈10 Gy). Results at SSD = 75 cm (D vs. E) should be interpreted cautiously because shorter SSD increases both DPP at the sample and instantaneous dose rate (IDR); we therefore attribute any additional effects in that setting primarily to IDR/DPP combined. Mechanistically, higher DPP could transiently increase intrapulse dose rate and reduce effective reoxygenation/repair within a pulse, but under clinical pulse widths, these effects are expected to be subtle and cell-line dependent; our findings are consistent with this and with each line’s α/β-implied fraction-size sensitivity.

When interpreted through the lens of α/β and intrinsic radiosensitivity, the results were biologically coherent, yet not uniformly conclusive. A549’s higher α/β was understood to predict limited fraction-size sensitivity and a smaller incremental benefit from time compression, whereas MCF-7’s lower α/β suggested a phenotype-dependent susceptibility to delivery timing in a mid-dose window, effects that were hinted at by the present data but were not established statistically. Importantly, the direction and magnitude of the survival fraction changes differed by cell line, indicating that FFF beam properties might have interacted with the tumor phenotype. This interpretation was consistent with our prior work in DU-145 prostate cancer cells [4], in which FFF at a higher nominal dose rate produced a measurable reduction in clonogenic survival under comparable, non-FLASH conditions, supporting the view that dose-rate effects could be tumor-type specific rather than universal. Considered together, the DU-145, A549, and MCF-7 datasets begin to populate a coordinated α/β-graded phenotype panel investigated under analogous VMAT-based FFF/FF irradiation conditions. DU-145 prostate carcinoma (intermediate radiosensitivity) [4] showed measurable dose-rate-dependent clonogenic suppression at ≥10 Gy single fractions; the present A549 lung carcinoma data (high α/β ≈ 12.4 Gy) show a selective ADR-associated effect centered around the 10 Gy single fraction; and the MCF-7 breast adenocarcinoma data (low α/β ≈ 4.1 Gy) show more consistent clonogenic decrements at 5–10 Gy under elevated ADR and DPP. Across these three radiobiologically distinct phenotypes, the qualitative pattern is convergent: FFF delivery effects are not uniform across tumor types but are modulated by intrinsic radiosensitivity, with the strongest signal located in dose windows where the underlying α/β phenotype is most informative. Although this phenotype panel remains limited and additional cell lines will be required to fully characterize the α/β gradient, the cross-study comparison provides converging evidence that any potential clinical benefit of FFF-mediated time compression is likely to be tumor-type specific rather than universally applicable. Concordant external evidence also showed heterogeneity: Nakano et al. [20] reported no survival change in A549 cells with high-rate FFF delivery, and comparable survival was reported across multiple lines under matched FFF versus FF conditions [9]. In addition, an RBE of approximately 1.0–1.06 with only small differences that disappeared at higher clinical dose rates has been measured [21]. In contrast, a trend toward reduced late survival at the highest FFF rate in V79 cells was observed, while glioblastoma stem-like cells were rate-insensitive, underscoring model-dependent responses [32]. Across several contrasts, WST cell viability analysis did not consistently mirror clonogenic survival (negative monotonic associations in Δ-space), particularly at 5–10 Gy, where clonogenic survival appeared to be the most delivery sensitive. The observed divergence was anticipated; the WST cell viability assay indicated mitochondrial and metabolic activity in the surviving cells, whereas clonogenic survival assessed the long-term ability to reproduce. Therefore, clonogenic survival was considered the main endpoint, with the WST cell viability assay serving as a supportive but not equivalent surrogate [26,41,42,43]. Our study was underpowered; therefore, the estimates were exploratory. The present experimental design did not permit complete physical isolation of ADR, DPP, and IDR as fully independent variables, since several delivery configurations simultaneously altered parameters such as beam energy, SSD, beam profile, and scatter conditions. Therefore, the observed radiobiological responses should be interpreted as being associated with specific delivery configurations rather than representing definitive isolated effects of individual physical parameters. In addition, direct sample-plane dosimetric verification at the cell monolayer level was not available for all delivery conditions; therefore, minor residual dosimetric differences between beam configurations cannot be fully excluded. The confirmation of the DPP, ADR, and IDR effects of FFF beams was judged to require larger replication and modeling that separated the aperture-dependent DPP at the sample from the nominal machine settings. As with other phantom-based in vitro irradiation systems, small dosimetric variations related to phantom composition and beam configuration cannot be completely excluded. IDR manipulation was implemented geometrically (SSD) under VMAT, and neither pulse-repetition frequency nor intrapulse width at the flask was measured; therefore, it was recommended that future work pair direct pulse-structure dosimetry at the sample with high-dose-aware modeling (e.g., USC-based EUD/TCP) and incorporate additional biological validation approaches where relevant. Given the exploratory design and limited per-group sample size, the statistical analyses were interpreted conservatively, with emphasis placed on overall response patterns and configuration-associated trends rather than isolated definitive effects. Considering the A549/MCF-7 data alongside the results of DU-145 cells, a reasonable clinical inference was made that FFF delivery at higher nominal dose rates could confer a superior therapeutic ratio relative to FF in certain phenotypes, provided that beam acceleration limited intrafraction repair without compromising plan quality, while consistent advantages across all histologies were unlikely. Most notably, within clinically non-FLASH conditions, a reproducible phenotype-contingent signal was observed: clonogenic survival showed the clearest delivery-rate dependence, selective and mid-dose-focused in A549 cells, but more pronounced at larger single fractions in MCF-7 cells, thereby establishing tumor biology as the dominant moderator of any incremental gain from FFF time compression in vitro.

## 5. Conclusions

The radiobiological responses associated with delivery configurations involving variations in ADR, DPP, and IDR were examined through single-fraction irradiations (5, 10, and 20 Gy) in two human cancer cell lines with different sensitivities to fraction size (A549 cells, working α/β ≈ 12.4 Gy; MCF-7 cells, working α/β ≈ 4.1 Gy). Across the planned comparisons, most per-dose analyses did not achieve statistical significance (α = 0.05). Where differences were observed-primarily within the 5–10 Gy range-they were inconsistent across doses and cell lines, thus interpreted as trends rather than conclusive effects. Specifically, pure DPP variations at a constant ADR (6FF vs. 6FFF at 600 MU·min^−1^) resulted in only modest changes in survival, whereas increases in ADR (time compression) and delivery at a reduced SSD (thereby increasing IDR and DPP at the sample) tended to produce greater, though still mostly non-significant, reductions in clonogenic survival. At 20 Gy, the inter-arm differences diminished, consistent with the near-saturation of lethal injury in a single fraction. WST cell viability (metabolic viability) and clonogenic (reproductive survival) assays frequently diverged, underscoring that the WST cell viability assay served as a supportive but non-equivalent surrogate for radiosensitivity, while clonogenic survival captured the long-term proliferative endpoint of primary interest. In personalized radiotherapy, it is recommended to evaluate FFF dose-rate strategies by tumor type/phenotype rather than expecting a uniform effect. Future research should focus on (i) expanding to additional tumor models (including prostate/DU-145 cells-like phenotypes, where prior data indicated significant rate dependence); (ii) increasing replication and power for per-dose contrasts; (iii) direct pulse-structure measurements at the sample (pulse-repetition frequency and intrapulse width) to quantify IDR/DPP in situ; and (iv) integrating high-dose-aware modeling with orthogonal endpoints (e.g., 3D spheroids, in vivo xenografts, immune, and normal-tissue readouts). Such studies are anticipated to elucidate when and for whom time-efficient FFF delivery meaningfully enhances tumor control while maintaining or improving normal tissue safety. Interestingly, in conditions that are not clinically classified as FLASH, a consistent signal linked to phenotype was observed. Clonogenic survival showed the most distinct dependence on delivery rate, being selective and concentrated at mid-doses in A549 cells, while more evident at higher single doses in MCF-7 cells. Thus, our current investigation highlighted tumor biology as the main factor influencing any additional benefits gained from FFF-mediated time compression in vitro. In summary, the radiobiological responses reported here should be interpreted as configuration-associated trends within an exploratory in vitro framework rather than as definitive isolated effects of any single delivery parameter. The dominant moderator of any potential FFF-related benefit appears to be tumor biology itself, and clinical translation will accordingly require tumor-type-specific evaluation rather than an assumption of universal effect.

## Figures and Tables

**Table 1:** Representative radiobiological parameters for A549 and MCF-7 cell lines derived from photon clonogenic survival data fitted with the linear-quadratic (LQ) model [27,28,29,30].

Cell Line	α (Gy^−1^)	β (Gy^−2^)	α/β (Gy)	SF_2_	D_10_ (Gy)	Radiosensitivity
A 549	~0.29	~0.023	~12.4 [27,28]	0.51	5.52	Higher α/β indicates reduced fraction-size sensitivity; more radiosensitive under conventional fractionation (lower SF_2_).
MCF-7	~0.16	~0.04	~4.1 [29,30]	0.62	5.85	Lower α/β indicates greater sensitivity to larger fraction sizes (greater benefit from hypofractionation). Relatively less radiosensitive at 2 Gy

**Table 2:** Experimental settings and labels. Setting I (SSD = 90 cm): A (6 MV FF, 600 MU·min^−1^), B (6 MV FFF, 600 MU·min^−1^), C (6 MV FFF, 1400 MU·min^−1^), and F (10 MV FFF, 2400 MU·min^−1^). Setting II (SSD = 75 cm): D (6 MV FF, 600 MU·min^−1^) and E (6 MV FFF, 600 MU·min^−1^). Labels A1–F3 indicate the prescribed dose (5/10/20 Gy). Abbreviations: DPP = dose per pulse;; FF = flattened; FFF = flattening-filter-free; MU = monitor units.

Experimental Settings	Label	Average Dose Rate MU/min	Dose per Pulse (DPP) ≈mGy/Pulse	Experimental Settings	Label	Average Dose Rate MU/min	Dose per Pulse (DPP) ≈mGy/Pulse
6ff_5Gy_6r_90	A1	600	0.28	6ff_5Gy_6r_75	D1	600	0.4
6ff_10Gy_6r_90	A2	600	0.28	6ff_10Gy_6r_75	D2	600	0.4
6ff_20Gy_6r_90	A3	600	0.28	6ff_20Gy_6r_75	D3	600	0.4
6fff_5Gy_6r_90	B1	600	0.78	6fff_5Gy_6r_75	E1	600	1.12
6fff_10Gy_6r_90	B2	600	0.78	6fff_10Gy_6r_75	E2	600	1.12
6fff_20Gy_6r_90	B3	600	0.78	6fff_20Gy_6r_75	E3	600	1.12
6fff_5Gy_14r_90	C1	1400	0.78	10fff_5Gy_24r_90	F1	2400	1.31
6fff_10Gy_14r_90	C2	1400	0.78	10fff_10Gy_24r_90	F2	2400	1.31
6fff_20Gy_14r_90	C3	1400	0.78	10fff_20Gy_24r_90	F3	2400	1.31

## Data Availability

All data generated or analyzed in this investigation are included in this article; additional data and materials will be made available by the corresponding author upon reasonable request.
